# Impact of patient-related factors on successful autologous fat injection laryngoplasty in thyroid surgical treated related unilateral vocal fold paralysis- observational study: Erratum

**DOI:** 10.1097/MD.0000000000019406

**Published:** 2020-02-14

In the article, “Impact of patient-related factors on successful autologous fat injection laryngoplasty in thyroid surgical treated related unilateral vocal fold paralysis- observational study”,^[1]^ which appears in Volume 99, Issue 1 of *Medicine*, the p-value for Stroboscopy rating form and Vocie handicap index-10 (sum) had the decimals in the wrong place. The correct table appears below.

**Table 2:**
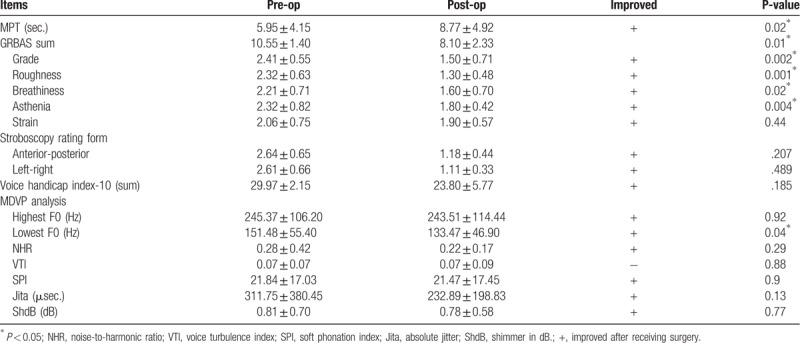
Outcomes before and after autologous fat injection laryngoplasty (n = 73).
